# Artificial Neural Network as a Tool to Predict Severe Toxicity of Anticancer Drug Therapy in Patients with Gastric Cancer: A Retrospective Study

**DOI:** 10.3390/diagnostics16020199

**Published:** 2026-01-08

**Authors:** Ugljesa Stanojevic, Dmitry Petrochenko, Irina Stanoevich, Ekaterina Pismennaya

**Affiliations:** 1 Ostroverkhov Clinical and Research Center for Oncology, 1 Eliseeva Str., Kislino Khutor, Ryshkovsky Selsovet, Kursk 305524, Russia; ugljesha@mail.ru; 2 Department of Oncology, Kursk State Medical University, 3 Karl Marx Str., Kursk 305004, Russia; 2849143@gmail.com; 3 Dedov National Medical Research Center of Endocrinology, 11 Dmitry Ulyanov Str., Moscow 117292, Russia; 4 Ministry of Healthcare of the Kursk Region, 6 Krasnaya sq., Kursk 305000, Russia; kurskzdrav@kursk.ru

**Keywords:** skeletal muscle index, gastric cancer, anticancer drug therapy, neural network, toxicity, oncology

## Abstract

**Background.** The aim of this study was to develop a predictive model of anticancer drug therapy toxicity in patients with gastric cancer. **Methods.** The retrospective study included 100 patients with stage II–IV gastric cancer who underwent 4 chemotherapy cycles. Initial significant toxicity factors included age, gender, height, body mass, body mass index, disease stage, skeletal muscle index (SMI), as well as plasma levels of trace elements (copper, zinc, selenium, manganese) and thyroid-stimulating hormone, cancer histology type and treatment regimen. The CTCAE v5.0 scale was employed to assess the severity of adverse events. Statistical analysis and building of mathematical neural network models were carried out in SPSS Statistics (v19.0). **Results.** Lower SMI values were associated with higher rates of toxicity-related complications of anticancer drug therapy (*p* < 0.05): leukopenia, hypoproteinemia, nausea, vomiting, cardiovascular events. Anemia, thrombocytopenia, hepatic cytolysis syndrome, nausea, diarrhea, constipation and stomatitis showed a weaker correlation with SMI. An increase in TSH was associated with higher rates of thrombocytopenia, nausea and vomiting. A decrease in Cu/Zn in plasma correlated with the severity of leukopenia and diarrhea, whereas Se/Mn showed an inverse correlation with the severity of anemia. **Conclusions.** Sarcopenia, abnormal thyroid status and imbalances in copper, zinc, selenium and manganese in blood plasma of patients with gastric cancer may be used as predictors of increased toxicity of anticancer drug therapy.

## 1. Introduction

Gastric cancer (GC) is a clinical entity with one of the highest prevalence and mortality rates in oncology [1,2]. Despite the decreasing prevalence of GC in developed countries, it has the fifth highest incidence among all cancers, and its cancer-specific mortality already ranks third [3,4]. While loss of appetite and weight up to cachexia are characteristic of a multitude of cancers, they are especially common in GC, reaching 57% [5]. Low muscle mass is thought to contribute to higher risks of toxicity during anticancer drug therapy [6]. Moreover, there is a robust body of evidence indicating fluctuations in trace elements during carcinogenesis, disease progression and toxicity-related complications associated with xenobiotic agents (including chemotherapy drugs). Copper, zinc, selenium and manganese are essential components of cellular antioxidation defense mechanisms and detoxification systems [7,8,9,10,11].

A growing body of evidence indicates that it is not the absolute values of trace element levels but rather the balance between specific trace elements (i.e., their ratios) that may serve as a more sensitive indicator of the functional status of the antioxidation system, inflammation and—as a result—vulnerability to toxic effects [12]. For example, the copper/zinc ratio has been acknowledged as the marker of systemic inflammation and oxidative stress in various chronic disorders [13]. Similar to that, the manganese/selenium ratio reflects the synergy of antioxidation systems: manganese superoxide dismutase (Mn-SOD) generates hydrogen peroxide, which is then converted to water by selenium-containing glutathione peroxidases (GPx). Imbalances between manganese and selenium may be indicative of incomplete breakdown of reactive oxygen species and higher risks of oxidative damage to tissues [14]. Nevertheless, the predictive roles of key trace element ratios (copper/zinc, selenium/manganese, copper/manganese, zinc/selenium) in toxicity-related complications of anticancer treatment remain poorly understood.

Anticancer drug treatment also affects the endocrine system, namely, the thyroid status, and alters the outcomes of treatment modalities associated with hormonal and metabolic shifts [15,16]. At the time of cancer detection, the majority of patients have chronic or newly diagnosed disorders of the thyroid gland, with some being incidental findings on diagnostic tests for cancer. However, thyroid gland disorders may also result from the treatment itself.

Mathematical models based on specific initial clinical signs and symptoms are widely employed to predict the course, relapses and outcomes (including death) of multiple disorders [17,18]. Current medical research often uses neural networks of various structures, such as recurrent neural networks [19,20], Kohonen self-organizing maps [21,22,23] and convolutional neural networks [24,25,26]. However, the classic example is the multilayer perceptron, a feedforward artificial multilayer network consisting of fully connected neurons [27,28]. This type of network features neurons of the previous layer linked by synapses to each neuron of the following layer [18]. Input layer neurons are fed mathematical signals describing prior medications, disease parameters, demographic data, etc. Each neuron in the input layer then forwards the data to each neuron of the hidden layer, where it is processed by a classic mathematical neuron. The process contains the following steps:

1. Each mathematical synapse is attributed a certain weight, which is multiplied by a value passing through the synapse.
2. Multiplied by the corresponding coefficients, the inputs of all the neuron synapses in the hidden layer are summed in the neuron’s adder and are represented by a single value.
3. The value obtained from the adder undergoes transformation via a series of mathematical functions to reduce it to fit a defined range.
4. The transformed and reduced to a range value of the adder is forwarded to all the neurons of the next layer of the neural network (next hidden layer or output layer) [18].

It is important to remember that initially, such a neural network is incapable of correct classification by itself; it has to be trained using a labeled dataset. The initial dataset is split into two unequal parts: the training set, accounting for 70%, and the test set, representing 30%. The former is used for neural network training itself, and the latter is used to evaluate the model’s performance, i.e., the quality of classification [18]. Thus, the information on all the objects of the training set passes through the neural network and undergoes synaptic weight correction to ensure that the number of correctly classified objects is as high as possible. Upon completion of training, the quality of classification is assessed by means of the test set [18].

The aim of this study was to develop a predictive model of anticancer drug therapy toxicity in patients with gastric cancer.

## 2. Materials and Methods

This retrospective study included treatment outcomes in 100 patients with locally advanced and metastatic gastric cancer who underwent chemotherapy in an inpatient department of Ostroverkhov Clinical and Research Center for Oncology (Kursk, Russia) from October 2021 to March 2023; anticancer drug therapy followed conventional chemotherapy protocols per national guidelines [29]. The decision regarding treatment was made on the basis of disease stage, Eastern Cooperative Oncology Group (ECOG) performance status, pathology report and comorbidities; FLOT was selected as neoadjuvant polychemotherapy (PCT) in resectable GC, and FOLFOX or XELOX were chosen for metastatic cancer. A thorough examination prior to treatment was carried out in an outpatient setting.

The inclusion criteria were male and female sexes, age ≥18 years, histologically verified stage II-IV gastric cancer (adenocarcinoma) diagnosed for the first time (according to the Eighth Edition AJCC Cancer Staging Manual), ECOG ≤2 and planned anticancer drug therapy for locally advanced (neoadjuvant chemotherapy) or metastatic gastric cancer (first-line therapeutic chemotherapy) according to national guidelines using a minimum of four cycles of platinum-based antineoplastics, taxanes and fluoropyrimidines (FLOT, FOLFOX, XELOX regimens with conventional protocols for neoadjuvant and therapeutic chemotherapy) and a signed informed consent form.

The exclusion criteria were age lower than 18 years, severe liver failure (Child-Pugh C) or renal failure (creatinine clearance less than 30 mL/min, calculated using the CKD-EPI equation), ECOG > 2, decompensated somatic disorders (diabetes mellitus, NYHA II-IV heart failure, uncontrolled arterial hypertension), disorders of consciousness preventing patients from understanding the treatment and study protocols, professional sports (owing to potentially altered physiological features and metabolism), pregnancy and lactation.

To measure the number of skeletal muscles and their loss rates, computed tomography was used (Discovery CT750 HD with accessories, GE Medical Systems, LLC, Chicago, IL, USA, slice thickness of 1.25 mm). Visualization tests followed the standard protocol from clinical guidelines on gastric cancer to assess potential tumor invasion and perform staging [29,30]. CT was carried out no earlier than one month prior to hospital admission and 2-3 weeks after 4 cycles of anticancer drug therapy.

The skeletal muscle index (SMI, cm^2^/m^2^) was calculated as the muscle area (cm^2^) in two subsequent axial sections at the level of the third lumbar vertebra (L3) divided by the patient’s height squared (m^2^). The threshold values for the SMI were 52.4 cm^2^/m^2^ for males and 38.5 cm^2^/m^2^ for females; lower SMIs were considered indicative of sarcopenia.

Plasma levels of trace elements (selenium, zinc, copper, manganese) were measured by means of mass spectrometry detection using the Varian 810-MS (Varian Inc., Palo Alto, CA, USA) inductively coupled plasma mass spectrometer prior to anticancer drug therapy and after 4 chemotherapy cycles. The spectrometer provides data on signal strength at a certain level of m/z [31].

Plasma levels of thyroid-stimulating hormone (TSH) were measured with an Immulite 2000 Siemens (Siemens Healthineers, Erlangen, Germany) automatic immunoassay system before radiological tests with iodine-based contrast agents or anticancer drug therapy.

The ECOG scale was used to assess patients’ overall condition, and the severity of adverse events was classified according to the National Cancer Institute Common Toxicity Criteria for Adverse Events (CTCAE v5.0) framework during anticancer drug therapy (after 4 cycles). The endpoint was any chemotherapy-induced hematological and nonhematological toxicity. Hematological toxicity included anemia (hemoglobin < 130 g/L in males and < 120 g/L in females), leukocytopenia (<4.0 × 109/L) and thrombocytopenia (<180 × 109/L). Nonhematological toxicity included hypoproteinemia (serum total protein less than 60 g/L), hepatic toxicity (liver enzymes (AST, ALT) 3.0 above the upper value of the normal range), nausea, vomiting, diarrhea, constipation, alopecia, hand–foot syndrome (HFS) and stomatitis (oral mucositis). Cardiovascular toxicity was assessed using the orthostatic test: all patients underwent blood pressure (BP) measurement using the Korotkov sphygmomanometer technique 5 min after lying still on their backs, as well as when standing 1 and 3 min after standing up independently. Blood pressure was measured prior to and after 4 cycles of anticancer drug therapy. Orthostatic hypotension was defined as a decrease in systolic blood pressure by 20 mm Hg (or by 30 mm Hg in patients with arterial hypertension) and/or diastolic blood pressure by at least 10 mm Hg or blood pressure below 90 mm Hg for 3 min after standing up. Other conditions interpreted as cardiovascular toxicity were the manifestation and/or progression of arterial hypertension, coronary heart disease, superficial and/or deep vein thrombosis in the lower extremities and venous thromboembolic events.

**Figure 1:**
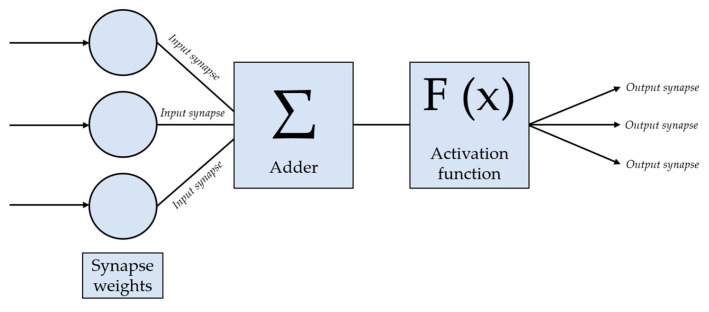
The general structure of the neural network.

**Figure 2:**
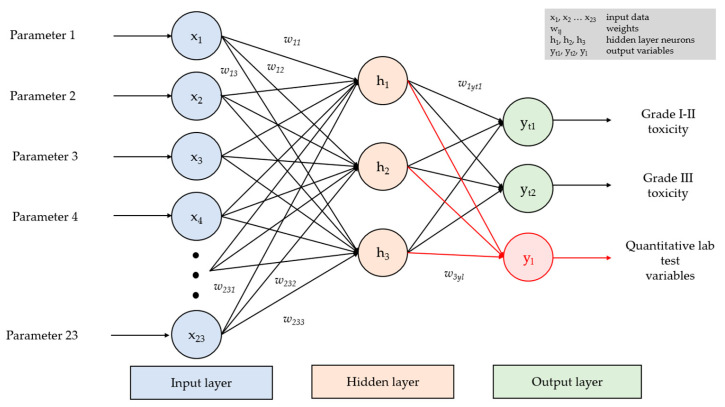
Perceptron architecture used in the current study.

In the current study, the multilayer perceptron was created using the Neural Networks module of the IBM SPSS Statistics software package (v19.0). The architecture of the neural network was not set manually but automatically during learning using built-in SPSS optimization algorithms. The architecture had a three-layer structure and included an input layer corresponding to the number of input parameters, a three-neuron hidden layer and an output layer for predictions. In automatic mode, SPSS selects the number of hidden layers and the number of neurons in them based on the properties of the training set. Optimization resulted in a three-layer configuration as it provided the best accuracy and reproducibility values. The schematic representation of the perceptron is given in Figure 1 and Figure 2.

The mathematical models of neural networks were built and tested on the basis of data from 100 patients with GC. A total of 13 mathematical models of neural networks were built to predict blood plasma levels of hemoglobin, white blood cells, platelets and serum total protein, as well as the severity of hepatic cytolysis syndrome, nausea, vomiting, diarrhea, constipation, stomatitis, alopecia, hand–foot syndrome and dysregulation of blood pressure after 4 cycles of anticancer drug therapy. The selection of a particular neural network was made on the basis of the best potential performance in regard to predicting adverse events of anticancer drug therapy in patients with GC.

Statistical analysis of the results was carried out using the SPSS Statistics (v19.0) software package. The Shapiro–Wilk test and Kolmogorov–Smirnov test were used to check for a normal distribution of quantitative variables. To describe quantitative variables with an abnormal distribution, the median, first quartile and third quartile (Me [Q1; Q3]) were employed. The normally distributed values are represented by the mean and mean-square deviation (M ± σ). Qualitative data is described as absolute values and percentages (*n*, %). Correlations between quantitative and ranked values were assessed using Spearman’s correlation coefficient. Quantitative variables were compared between two groups via Student’s *t*-test. A *p*-value of <0.05 was chosen as the threshold for statistical significance.

## 3. Results

Patient data is given in Table 1. Among the 100 patients included, 76 (76.0%) were male and 24 (24.0%) were female. SMI values before treatment were indicative of sarcopenia in 92 (92.0%) patients (75 (98.7%) males and 17 (70.8%) females).

No signs of deficiency or excess of copper, zinc, selenium or manganese were detected: the trace element levels remained within normal ranges before and after 4 courses of polychemotherapy. When absolute values were analyzed, while no deviation from the normal ranges was detected, a statistically significant (*p* < 0.05) decrease in copper and zinc levels, as well as an increase in selenium and manganese were noted by the fifth chemotherapy cycle. Statistically significant changes in the ratios of trace elements (copper/zinc, selenium/manganese, copper/manganese and selenium/zinc) were also observed after four cycles of anticancer drug therapy.

Histological examination revealed high-grade adenocarcinoma in 56 (56.0%) patients (42 (75.0%) males and 14 (25.0%) females) and low-grade adenocarcinoma in 44 (44.0%) patients (34 (77.3%) males and 10 (22.7%) females), *p* > 0.05.

Toxicity-related complications after 4 cycles of anticancer drug therapy are outlined in Table 2. Clinically relevant peripheral polyneuropathy was not observed after 4 cycles of anticancer drug therapy because it usually manifests later, and its cumulative effects take more time to become apparent.

In 91 (91.0%) patients, toxicity during anticancer drug therapy did not warrant cessation of/pause in treatment or initial anticancer agent dose reduction; these patients were successfully managed with conventional symptomatic therapy. A pause in therapy and dose reduction were required in two patients due to thromboembolic events, grade III anemia and leukopenia, as well as in patients with grade III hand–foot syndrome. Grade IV-V toxicity was not observed during anticancer drug therapy.

Cardiovascular toxicity started developing during the first cycle of anticancer drug therapy. Arterial hypertension as a comorbidity was noted in 68 (68.0%) patients, of whom 28 patients (41%) had stopped antihypertensive treatment several months before the start of chemotherapy owing to a shift toward hypotension. Six (6.0%) patients had stable coronary artery disease. Apart from the dysregulation of blood pressure found in 38 (38.0%) patients, one patient developed coronary heart disease in the form of acute anteroseptal ST-elevation myocardial infarction after 4 cycles of chemotherapy, and one patient progressed to FC III stable angina, which necessitated more intensive treatment with antianginal agents. Moreover, there were 4 cases of acute deep vein thrombosis, one of which resulted in segmental pulmonary thromboembolism (in the left pulmonary artery).

The strength and direction of the relationship between two variables represented by Spearman’s correlation coefficient (r) are given in Table 3. TSH levels had a statistically significant moderate correlation with the severity of thrombocytopenia, nausea and vomiting after 4 cycles of anticancer drug therapy, and the SMI showed a moderate correlation with the toxic effects of chemotherapy (leukopenia, hypoproteinemia, nausea and vomiting) and an average statistically significant correlation with cardiovascular events.

A moderate inverse statistically significant correlation was revealed between the copper/zinc ratio before treatment and the severity of leukopenia after 4 chemotherapy cycles (r = −0.331; *p* < 0.05). A weak inverse statistically significant correlation (*p* < 0.05) was observed between the selenium/manganese ratio before treatment and anemia severity after 4 chemotherapy cycles (r = −0.211). A moderate inverse (r = −0.331) statistically significant (*p* < 0.05) correlation was noted between the copper/zinc ratio before treatment and the severity of diarrhea after 4 chemotherapy cycles. A weak inverse statistically significant correlation (*p* < 0.05) was detected between the copper/manganese ratio before treatment, the zinc/selenium ratio before treatment, the copper/zinc ratio after 4 cycles and the severity of diarrhea after 4 chemotherapy cycles (r = −0.206; r = −0.199; r = −0.241, respectively).

The properties of neural networks used to predict toxic complications of anticancer drug therapy in patients with gastric cancer are given in Table 4. All the developed models employed the same neural network architecture, which included three layers: input, hidden and output. The input layer was comprised of 23 neurons, corresponding to clinical, demographic and laboratory data and patients’ history. The hidden layer included three neurons. The output layer contained one neuron if predictions were made regarding quantitative blood test parameters and two neurons if predictions were made regarding the severity of toxicity-related complications.

In each model, hidden layer neurons were activated with the hyperbolic tangent activation function (for analog activation of all neurons and mapping a set of all real numbers into range sets). The output layer neurons were activated with Softmax (to normalize the network output to a probability distribution over the predicted output classes). Categorization of the training set was performed via machine learning algorithms. The percentage of correct classifications reflects the accuracy of the predictive model.

Table 5, Table 6 and Table 7 list the predictors included in the models that were selected by the neural networks during training. The predictive value of the models only reached clinically significant ranges if all the selected predictors were included.

The highest prognostic values in neural network models that predict toxic complications of anticancer drug therapy in patients with GC were shown by sex, age, height, body mass, body mass index (BMI), body surface area (BSA), functional status according to the Karnovsky scale, SMI, TSH and levels of trace elements in blood plasma (copper, zinc, selenium, manganese). Conversely, the least reliable predictors were disease stage, treatment regimen, tumor histology and type 2 diabetes mellitus.

## 4. Discussion

The loss of skeletal muscles is an age-related physiological process that can be exacerbated by systemic disorders, especially those that result in limited physical activity. A lower SMI is associated with higher rates of toxic complications of anticancer drug therapy, with no difference between sexes (*p* > 0.05): leukopenia, hypoproteinemia, nausea, vomiting and cardiovascular events. While anemia, thrombocytopenia, hepatic cytolysis syndrome, nausea, diarrhea, constipation and stomatitis (oral mucositis) are notable side effects of chemotherapy, they are relatively weakly correlated with SMI, which likely stems from the small sample of patients. No differences in the histological types of GC between sexes were noted. The data obtained in the current study (including sex, age and toxicity type) corresponds to the outcomes of other trials exploring the link between sarcopenia and anticancer drug therapy toxicity [32,33,34,35,36].

TSH levels turned out to be strong predictors of adverse events associated with anticancer drug therapy. Higher TSH levels were linked to higher rates of toxicity-related complications of drug therapy, such as thrombocytopenia, nausea and vomiting. Although adequate assessment of the thyroid gland and status prior to the initiation of anticancer drug therapy is an important step toward satisfactory outcomes in patients with cancers, guidelines still fail to recommend it, with the exception of immune checkpoint inhibitors. The toxic effects of anticancer drug therapy may manifest as symptoms characteristic of hypo- or hyperthyroidism. Higher or lower basal metabolic rates, in turn, obviously impact drug metabolism. Taken together, altered thyroid status parameters may result in erroneous dosing, i.e., lowering the dose or even halting therapy altogether [15,16]. For example, signs and symptoms of hypothyroidism, such as fatigue, weakness, depression, memory loss, cold intolerance and changes in the cardiovascular system, may be falsely attributed to cancer itself or drug therapy. O.P Hamnvik et al. recommended routine checks of the functional activity of the thyroid gland in patients who receive cytotoxic agents [16]. Notably, several conventional cytostatic drugs (such as lomustine, vincristine and cisplatin) have been proven to affect the thyroid gland in vitro with no obvious clinical signs [37,38,39]. 5-Fluorouracil, a widely used anticancer agent, increases thyroxin and triiodothyronine levels, while neither changes in TSH nor clinical signs of hyperthyroidism are observed [40].

For several decades, altered levels of trace elements have been known to cause DNA/cell damage and oxidative stress, with both processes triggering malignant transformation [41,42,43,44]. Monitoring the plasma levels of copper, zinc, manganese and selenium in patients with GC who receive anticancer drug therapy has potential clinical applications. The copper and zinc levels decreased after 4 cycles of polychemotherapy, which may stem from increased demand or loss as a direct side effect of anticancer agents. Such changes to zinc levels have been reported to be associated with neurotoxicity in patients with oncologic disorders of the female reproductive system who received taxane-based chemotherapy [45]. Higher selenium and manganese levels after 4 cycles of chemotherapy warrant further research. This may be linked to the decreased bioavailability of these trace elements in tissues in response to polychemotherapy or changes in their metabolic rates. The selenium/manganese ratio reflects the balance of the key components of the antioxidation system: selenoproteins (GPx) and Mn-SOD, which neutralizes superoxide in mitochondria. A relative decrease in selenium levels may indicate insufficient GPx-mediated protection of erythrocyte membranes from lipid peroxidation, leading to hemolysis. Moreover, oxidative stress in erythroblast mitochondria, poorly controlled owing to selenium/manganese imbalance, may impair erythropoiesis [46]. In the PC3 cell line, a combination of manganese and docetaxel significantly increased the cytotoxic effect, resulting in lower doses of docetaxel and potentially lower complication rates [47].

The obtained data revealed statistically significant correlations with hematological and gastrointestinal complications. Low ratios of trace elements (copper and zinc) were associated with severe leukopenia and diarrhea after 4 chemotherapy cycles, and lower selenium/manganese ratios showed a statistically significant correlation with increased severity of anemia. Zinc is vital for cell proliferation (including white blood cells and enterocytes), protein synthesis and proper functioning of the immune system, and its relative deficiency impedes reparation of the mucous membrane of the gastrointestinal tract and white blood cell regeneration [46]. Experimental works showed that zinc deficiency exacerbates mucositis induced by 5-fluorouracil and addition of zinc to the treatment protocol yielded protective effects [48]. Clinical trials also linked low zinc levels to the severity of gastrointestinal toxicity [49]. While the antagonism between copper and zinc absorption in the gastrointestinal tract is a well-known phenomenon, both trace elements are found in the Cu/Zn superoxide dismutase (Cu/Zn-SOD), which plays a role in the antioxidation system and detoxification of xenobiotics. Cu/Zn imbalance has a direct impact on Cu/Zn-SOD activity, which has been proven in in vitro trials and clinical studies on various clinical entities [50].

While a vast body of research has been dedicated to measuring the levels of trace elements in patients with GC in various biological samples, papers exploring the impact of imbalances in trace elements on adverse events linked to anticancer drug therapy, to the best of our knowledge, are nonexistent.

The study has several limitations that may impact the interpretation of the results. First of all, the small sample decreases the statistical significance and limits generalizability. If the sample is small, machine learning techniques may exhibit more sensitivity to individual patient parameters, potentially leading to overlearning and overestimation of the prognostic accuracy of the models. The limited sample was a consequence of the retrospective nature of the study and strict inclusion criteria. The only patients who were included in the study were those who had undergone all the diagnostic tests discussed in the article.

This was a single-center trial, which limits the variability of clinical and demographic data, anticancer treatment regimens and supportive care. This may decrease the versatility of the developed models in other clinical environments with alternative treatment protocols.

Another limitation is that the trial did not include all the possible factors that may influence toxicity-related complications of anticancer treatment. In particular, pharmacogenetic properties, concomitant medication and details of the nutritional status were not accounted for, which may have influenced the accuracy of prognostic models for select toxic effects.

The employed neural network models were assessed by means of internal validation, without an external check using an independent set. The lack of external validation limits overall assessment and reproducibility of the models.

Multi-center trials on larger and more representative groups of patients are warranted. Future studies may also benefit from a wider range of clinical, laboratory and molecular parameters. The developed neural network models that predict the toxic effects of anticancer drug therapy in patients with GC may find their place in clinical practice. Notably, in the neural network models, the key predictors of toxicity-related complications of drug therapy were age, SMI before treatment and plasma levels of trace elements and TSH.

One of the key advantages of the developed neural network models is their ability to take into account objective lab test results and diagnostic tests as a whole (not isolated data), which confirms the efficacy of the models and their potential for predicting toxicity-related complications of anticancer treatment. Prognostic accuracy is the result of a complex analysis of a multitude of interconnected factors, each of which has no predictive value by itself. This highlights the importance of investigations and diagnostic tests for the models as they improve prognostic accuracy. The high accuracy of the predictions on the basis of objective parameters indicates that the developed neural network algorithms are reliable.

Neural-network-based analysis and suggested parameters yielded high-accuracy predictions of hematological toxicity and hypoproteinemia as complications of anticancer drug therapy for GC.

## 5. Conclusions

Sarcopenia, abnormal thyroid status and imbalances in plasma levels of trace elements in patients with gastric cancer may act as predictors of a greater severity of toxicity-related complications of anticancer drug therapy.

The results of the current work offer clear applications in clinical practice. The developed multilayer-perceptron-based mathematical models capable of predicting severe toxic effects associated with anticancer drug therapy for gastric cancer completely follow the principles of translational medicine, which strives to translate basic science and fundamental research into actual medical practice [51]. The developed mathematical models for predicting hematological toxicity and hypoproteinemia can be readily incorporated into the daily clinical practice of oncologists and chemotherapists; the models predict toxicity-related complications of anticancer drug therapy in patients with gastric cancer on the basis of clinical data, laboratory findings and investigations prior to the commencement of anticancer drug therapy.

## Figures and Tables

**Table 1:** Clinical and demographic data of the patients (*n* = 100) included in the study.

Parameter	Males and Females	Males	Females
Gender, absolute value (%)	-	76 (76.0%)	24 (24.0%)
Age, years M ± σ, Me [Q1; Q3]	64.50 ± 8.85	64.17 ± 8.19	66 [63.0; 69.75]
Anthropometric data, M ± σ			
- height, m	1.68 ± 0.09	1.71 ± 0.069	1.58 ± 0.063
- mass, kg	64.63 ± 14.64	66.12 ± 14.22	66.17 ± 11.09
- BMI, kg/m^2^	22.98 ± 4.92	22.65 ± 4.23	26.35 ± 3.96
SMI, prior to treatment (M ± σ), cm^2^/m^2^	36.21 ± 6.86	38.78 ± 6.73	34.69 ± 6.59
SMI, after 4 treatment cycles (M ± σ), cm^2^/m^2^	32.31 ± 6.34	34.55 ± 6.49	31.79 ± 6.64
ΔSMI, (Me [Q1; Q3]), cm^2^/m^2^	2.39 [1.38; 5.64]	3.20 [1.42; 6.76]	2.28 [1.75; 4.03]
Plasma levels of trace elements prior to treatment, M ± σ; Me [Q1; Q3]			
- Copper, μg/L	1008.54 ± 245.80	975.59 ± 150.64	1104.70 ± 270.49
- Zinc, μg/L	778.74 ± 166.20	807.19 ± 156.55	773.07 ± 140.02
- Selenium, μg/L	130.66 [110.78; 152.51]	141.42 [115.71; 148.51]	134.08 [112.19; 150.34]
- Manganese, μg/L	1.07 [0.90; 1.36]	1.17 [0.91; 1.28]	1.13 [0.97; 1.19]
Plasma levels of trace elements after 4 treatment cycles, M ± σ; Me [Q1; Q3]			
- Copper, μg/L	970.18 ± 259.11	941.16 ± 264.06	944.29 ± 332.16
- Zinc, μg/L	727.20 ± 152.62	743.72 ± 157.27	718.95 ± 185.09
- Selenium, μg/L	161.78 ± 19.23	162.28 ± 15.86	161.53 ± 20.43
- Manganese, μg/L	1.14 [0.90; 1.36]	1.2 [0.92; 1.18]	1.37 [0.93; 1.31]
TSH, μIU/mL Me [Q1; Q3]; M ± σ	1.09 [0.74; 1.93]	1.63 ± 2.70	1.82 [0.87; 2.64]
Functional status, absolute value (%)			
- ECOG 0	23 (23.0%)	18 (24.7%)	5 (17.9%)
- ECOG 1	64 (64.0%)	43 (58.9%)	21 (75.0%)
- ECOG 2	14 (14.0%)	12 (16.4%)	2 (7.1%)
Disease stage, absolute value (%)			
- I	-	-	-
- II	18 (18.0%)	13 (17.1%)	5 (20.8%)
- III	43 (43.0%)	31 (40.8%)	12 (50.0%)
- IV	39 (39.0%)	32 (42.1%)	7 (29.2%)
T, absolute value (%)			
- T1	-	-	-
- T2	9 (9.0%)	5 (6.6%)	4 (16.7%)
- T3	60 (60.0%)	43 (56.6%)	17 (70.8%)
- T4	31 (31.0%)	28 (36.8%)	3 (12.5%)
N, absolute value (%)			
- N0	4 (4.0%)	2 (2.6%)	2 (8.3%)
- N1	58 (58.0%)	45 (59.2%)	13 (54.2%)
- N2	35 (35.0%)	27 (35.6%)	8 (33.3%)
- N3	3 (3.0%)	2 (2.6%)	1 (4.2%)
M, absolute value (%)			
- M0	61 (61.0%)	44 (57.9%)	17 (70.8%)
- M1	39 (39.0%)	32 (42.1%)	7 (29.2%)
Treatment regimen, absolute value (%)			
- FLOT	61 (61.0%)	44 (57.9%)	17 (70.8%)
- FOLFOX	25 (25.0%)	20 (26.3%)	5 (20.8%)
- XELOX	14 (14.0%)	12 (15.8%)	2 (8.4%)
Comorbidity, absolute value (%)			
- Coronary heart disease	6 (6.0%)	4 (5.3%)	2 (8.3%)
- Arterial hypertension	68 (68.0%)	45 (59.2%)	23 (95.8%)
- Type 2 diabetes	6 (6.0%)	4 (5.3%)	2 (8.3%)
- COPD, including asthma	18 (18.0%)	16 (21.0%)	2 (8.3%)

**Table 2:** Toxicity after 4 cycles of anticancer drug therapy.

Toxic Effect	Toxicity Grade, Absolute Value (%)		
	I–II	III	IV
Nausea	69 (69.0%)	6 (6.0%)	-
Vomiting	34 (34.0%)	5 (5.0%)	-
Diarrhea	48 (48.0%)	10 (10.0%)	-
Constipation	8 (8.0%)	1 (1.0%)	-
Stomatitis	13 (13.0%)	-	-
Anemia	48 (48.0%)	2 (2.0%)	-
Leukopenia	29 (29.0%)	2 (2.0%)	-
Thrombocytopenia	8 (8.0%)	-	-
Hepatic cytolysis syndrome	9 (9.0%)	-	-
Hypoproteinemia *	26 (26.0%)		
Alopecia **	100 (100.0%)	-	-
Hand–foot syndrome	15 (15.0%)	2 (2.0%)	-
Peripheral polyneuropathy *	-	-	-
Blood pressure dysregulation *	38 (38.0%)		
Myocardial infarction *	1 (1.0%)		
Angina *	1 (1.0%)		
Acute deep vein thrombosis *	3 (3.0%)		
Pulmonary embolism *	1 (1.0%)		

* Classification according to CTCAE is unavailable. ** Only grades I and II available for alopecia in the CTCAE framework.

**Table 3:** Correlation coefficients between TSH, SMI and the severity N = 100.

	TSH	SMI Before Treatment	SMI After 4 Cycles of PCT	ΔSMI
Thrombocytopenia after 4 cycles of PCT	−0.344 * 0.021 **	- ***	-	-
Nausea after 4 cycles of PCT	−0.335 0.038	−0.294 0.031	-	-
Vomiting after 4 cycles of PCT	−0.304 0.018	−0.304 0.036	-	-
Hypoproteinemia after 4 cycles of PCT	-	−0.335 0.003	−0.297 0.010	-
Leukopenia after 4 cycles of PCT	-	-	−0.307 0.030	-
Systolic blood pressure	-	0.665 0.001	0.634 0.001	0.648 0.001
Diastolic blood pressure	-	0.686 0.001	0.548 0.001	0.585 0.001
Systolic blood pressure (orthostatic test, first minute)	-	0.548 0.001	0.664 0.001	0.594 0.001
Diastolic blood pressure (orthostatic test, first minute)	-	0.526 0.001	0.567 0.001	0.543 0.001

* correlation coefficient. ** statistical significance (*p*-value). *** no statistical significance (*p* ≥ 0.05). Interpretation of correlation coefficients: 1. Very strong (0.9 < r < 1.0). 2. Strong (0.7 < r < 0.89). 3. Average (0.5 < r < 0.69). 4. Moderate (0.3 < r< 0.49). 5. Weak (0.2 < r < 0.29). 6. Very weak (0.0 < r < 0.19).

**Table 4:** Prognostic model parameters for blood plasma hemoglobin, white blood cell count, platelet count, total protein and the severity of cytolysis, nausea, vomiting, diarrhea, constipation, stomatitis, alopecia, HFS and blood pressure dysregulation.

Prognostic Model for	Proportion of All Correct Classifications in the Training Set, %	Proportion of All Correct Classifications in the Testing Set, %	Area Under the ROC-Curve
Blood plasma hemoglobin	98.5	97.3	0.974
Blood plasma white blood cell count	92.5	91.2	0.910
Blood plasma platelet count	91.6	90.4	0.913
Serum total protein	94.9	91.2	0.917
Severity of cytolysis	84.3	83.3	0.842
Severity of nausea	59.5	52.4	0.675
Severity of vomiting	46.5	52.8	0.641
Severity of diarrhea	65.8	59.3	0.716
Severity of constipation	66.1	80.5	0.675
Severity of stomatitis	75.0	78.1	0.739
Severity of alopecia	87.0	84.0	0.854
Severity of HFS	83.1	73.8	0.821
Severity of blood pressure dysregulation	66.7	56.0	0.579

**Table 5:** Contribution of predictors into prognostic models for the plasma levels of hemoglobin, white blood cells, platelets and total protein.

Predictor	Normalized Weight (%)			
	Blood Plasma Hemoglobin	Blood Plasma White Blood Cell Count	Blood Plasma Platelet Count	Serum Total Protein
Gender	32.1%	11.8%	14.8%	**76.3%**
Age	**100.0%**	20.1%	**98.1%**	**94.9%**
GC stage	23.2%	20.2%	47.3%	41.1%
Treatment regimen	23.5%	20.5%	38.6%	25.1%
Height	41.2%	33.7%	42.8%	40.4%
Body mass	48.3%	35.9%	44.8%	80.0%
BMI	**89.3%**	**100.0%**	**87.1%**	**98.9%**
Karnovsky scale	**85.1%**	38.4%	8.9%	45.1%
BSA	39.7%	**87.4%**	17.4%	**89.5%**
Histological tumor type	5.7%	26.3%	30.9%	61.2%
Type 2 diabetes mellitus	12.6%	7.4%	8.2%	10.0%
SMI before treatment	8.1%	33.2%	**74.3%**	**100.0%**
Copper before treatment	20.1%	**81.1%**	**85.0%**	20.9%
Zinc before treatment	**89.2%**	48.4%	30.2%	**75.5%**
Selenium before treatment	**81.1%**	18.9%	**100.0%**	38.8%
Manganese before treatment	**85.2%**	**58.9%**	**60.9%**	15.4%
TSH before treatment	**91.3%**	34.3%	**98.9%**	41.9%

Values in bold represent substantial contributions.

**Table 6:** Contribution of predictors into prognostic models for the severity of cytolysis, nausea, vomiting, diarrhea and constipation.

Predictor	Normalized Weight (%)				
	Cytolysis	Nausea	Vomiting	Diarrhea	Constipation
Gender	8.5%	20.6%	16.2%	17.4%	13.7%
Age	**65.6%**	**81.4%**	**100.0%**	57.7%	31.8%
GC stage	20.8%	33.4%	21.5%	16.0%	20.7%
Treatment regimen	50.2%	25.2%	20.3%	15.3%	24.0%
Height	35.9%	**85.0%**	42.0%	**61.5%**	30.9%
Body mass	**79.8%**	51.2%	**75.1%**	100.0%	67.4%
BMI	**100.0%**	**100.0%**	38.5%	25.2%	45.7%
Karnovsky scale	70.2%	38.4%	58.9%	18.1%	**98.1%**
BSA	**79.2%**	**71.8%**	17.4%	19.6%	27.8%
Histological tumor type	2.8%	19.1%	18.3%	14.8%	12.3%
Type 2 diabetes mellitus	14.8%	19.9%	16.4%	7.3%	1.5%
SMI before treatment	**97.5%**	**98.7%**	24.4%	94.1%	38.6%
Copper before treatment	25.7%	53.6%	26.0%	**98.2%**	38.3%
Zinc before treatment	57.7%	**73.0%**	**75.4%**	57.0%	11.2%
Selenium before treatment	25.1%	**79.0%**	**72.1%**	57.4%	**68.0%**
Manganese before treatment	38.3%	43.2%	59.5%	16.3%	**100.0%**
TSH before treatment	58.5%	**62.0%**	58.0%	**60.8%**	**78.5%**

Values in bold represent substantial contributions.

**Table 7:** Contribution of predictors into prognostic models for the severity of stomatitis, alopecia, hand–foot syndrome and blood pressure dysregulation.

Predictor	Normalized Weight (%)			
	Stomatitis	Alopecia	HFS	BP Dysregulation
Gender	17.0%	34.9%	18.2%	19.3%
Age	47.5%	31.4%	28.0%	**100.0%**
GC stage	35.3%	34.0%	16.3%	10.8%
Treatment regimen	40.3%	32.0%	19.4%	34.8%
Height	58.9%	39.0%	7.4%	**84.2%**
Body mass	35.1%	**100.0%**	**100.0%**	30.8%
BMI	16.0%	**97.4%**	18.4%	17.8%
Karnovsky scale	**80.2%**	31.4%	36.9%	**68.1%**
BSA	**78.4%**	18.4%	17.4%	59.2%
Histological tumor type	16.2%	30.4%	10.6%	30.5%
Type 2 diabetes mellitus	5.7%	24.3%	10.6%	12.1%
SMI before treatment	58.4%	48.4%	7.5%	**74.1%**
Copper before treatment	**80.1%**	32.1%	17.9%	32.6%
Zinc before treatment	**88.2%**	37.4%	48.1%	57.0%
Selenium before treatment	57.2%	**57.9%**	10.5%	55.8%
Manganese before treatment	**100.0%**	**59.7%**	**57.3%**	7.1%
TSH before treatment	43.8%	33.3%	53.1%	**78.1%**

Values in bold represent substantial contributions.

## Data Availability

Data is contained within the article.

## Abbreviations

The following abbreviations are used in this manuscript:

BMI	Body mass index
BP	Blood pressure
BSA	Body surface area
COPD	Chronic obstructive pulmonary disease
CTCAE	Common toxicity criteria for adverse events
ECOG	Eastern Cooperative Oncology Group
FC	Functional class
GC	Gastric cancer
GPx	Glutathione peroxidases
HFS	Hand–foot syndrome
PCT	Polychemotherapy
SMI	Skeletal muscle index
SOD	Superoxide dismutase
TSH	Thyroid-stimulating hormone
